# Modification in aortic arch replacement surgery

**DOI:** 10.1186/s13019-017-0689-y

**Published:** 2018-02-12

**Authors:** Feng Gao, Yongjie Ye, Yongheng Zhang, Bo Yang

**Affiliations:** 1 Department of Cardiovascular Surgery, Xiangya Haikou Hospital of Middle South University, Haikou Municipal Hospital, Haikou Vascular Disease Research Institute, The No. 43 People Road, Haikou City, 570208 China; 2 Department of Cardiovascular Surgery, SuiNing Central Hospital, SuiNing City, China

**Keywords:** Hybrid procedure, Debranch procedure, Aortic arch replacement, Aortic dissection type A

## Abstract

**Objective:**

We modified the conventional aortic arch replacement procedure to avoid circulation arrest and a prolonged extracorporeal circulation time, especially in cases of acute aortic dissection. We herein present our experience with a modified branch-first approach to acute aortic dissection, with anastomosis of the supra aortic vessels prior to commencing cardiopulmonary bypass.

**Methods:**

Since 2012, 41 patients (aortic dissection, 36; arch aneurysm, 5) have undergone the modified procedure. Procedurally, the implanted graft was used as a landing zone for second-stage endovascular stent-graft deployment intended to manage the residual descending dissection. Antegrade and retrograde systemic perfusion was instituted during cardioplegic arrest. The brain was actively perfused via the graft throughout the procedure.

**Results:**

Arch replacement surgery could generally be completed within approximately 4 h. During a 2-year period of aortic dissection or arch aneurysm treatment, only four anastomoses were required during the first stage of operation: two in the aorta, and one each in the innominate and left common carotid arteries. No patient died of surgical causes, and no stent grafts were deployed into the false lumen, a characteristic of procedures using traditionally antegrade deployment.

**Conclusion:**

We recommend that our procedure for acute aortic dissection be performed in two stages (graft replacement first and stent graft deployment second), particularly for patients underwent preoperative hypotesion. If malperfusion syndrome still exists after graft replacement, stent graft should be deployed in one stage. The arch aneurysm can be treated in one stage because there is no concern about false lumen deployment.

**Electronic supplementary material:**

The online version of this article (10.1186/s13019-017-0689-y) contains supplementary material, which is available to authorized users.

We modified the conventional aortic arch replacement procedure, especially for acute aortic dissection, to avoid circulation arrest and a prolonged extracorporeal circulation time, which have been identified as independent risk factors for morbidity and mortality [1, 2].

## Surgical techniques

Preparation: We recommend performing this procedure in a hybrid operation room. Bilateral cerebral oxygen saturation and invasive artery pressure in the bilateral upper limbs were monitored continuously during the operation.

### Operation steps (Additional file 1: Video 1)

After performing sternotomy and opening the pericardium, the tissues were dissected free as much as possible to expose the aortic arch and supra-arch branches. The adventitia was reserved as much as possible to facilitate suturing of the fragile arteries in an acute setting. The patient was subsequently heparinised.


Additional file 1:Video 1 showed the procedural steps of simplified arch replacement approach. (MP4 286720 kb)


We used a fine paediatric vascular clamp, such as a Pilling clamp 354,486, to fully close the left carotid artery (LCA) and thus avoid secondary injury to the intima. We usually sutured the intima to the adventitia, which had been severely torn, using a 7–0 prolene suture and mattress-suturing and added a pericardial strip outside the carotid artery. Next, we completed the end-to-side anastomosis between the branch and LCA using a 6–0 prolene suture.

We then measured the length of the second branch of the aortic graft (28 mm; Interguard, Maquet, France) and clipped it, and clamped and transected the innominate artery and trimmed the end to prepare for anastomosis. We anastomosed the graft branch and innominate artery in an end-to-end manner using a 5–0 prolene suture, and completed the end-to-side anastomosis between the branch and left common carotid artery. These end-to-side and end-to-end anastomoses are key to understanding how to actively perfuse the brain. Specifically, this method does not cause brain circulatory arrest of blood flow from the carotid to the innominate artery during the procedure, yet it allows the aorta to be clamped near the carotid artery. The proximal end of the innominate artery does not require suturing if the segment has been severely dissected; rather, clamping can be performed until the aorta is closed.

The left femoral artery was exposed and cannulated using a cannula connected to the cardiopulmonary bypass to yield retrograde artery perfusion. A hybrid operating room can confirm that the cannula has been placed in the true aortic lumen. We inserted a two-stage cannula in the right atrium, and established another artery cannula branched from the artery end of cardiopulmonary bypass using a Y-shaped connector to the perfusion branch of the graft. Accordingly, the aorta was perfused in both an antegrade and retrograde manner, which allowed adequate perfusion of the viscera, even in the presence of preoperative malperfusion syndrome. The cardiopulmonary bypass was established, and the blood was cooled to 28 °C.

Once the blood temperature reached 32 °C, the aorta was clamped near the left common carotid artery. The ascending aorta was opened, and cardioplegia fluid was perfused into the left and right coronary arteries to induce cardiac arrest. We trimmed the proximal end of the aorta and reinforced the aortic wall with two Dacron strip patches using the sandwich technique. The distal end of the aorta was treated in the same manner. The distal anastomosis of the aorta and graft was achieved with a 3–0 prolene suture, after which we released the clamp distal to the graft to determine whether any bleeding would occur. We found that it was safer and more efficient to wrap the anastomosis with a strip patch than to use sutures. The proximal anastomosis of the aorta with the graft was performed in the same manner.

Finally, the patient was rewarmed and the heart beat recovered. The root of the left common carotid artery was closed using a 5–0 prolene suture to prevent retrograde blood flow into the false lumen. From that point, the aortic dissection was changed from type A to type B. Extracorporeal circulation was restored in a stepwise manner to complete the procedure. Angiography revealed the remaining distal dissection. Endovascular repair was not immediately required except in cases of low perfusion syndrome or a threatened rupture.

Two weeks later, the patient returned to the operating room, where a femoral approach to expose the femoral artery was performed under local infiltration anaesthesia. Once the catheter reached the ascending aorta, a superstiff guidewire (Lunderquist, COOK, US) was introduced for stent graft deployment. Finally, a 20-cm-long stent graft (34 × 200 mm; Tag, GORE, US) was deployed near the second graft branch orifice.

## Comments

Morbidity and mortality can be improved by reducing blood perfusion; the durations of general anaesthesia, cardiopulmonary bypass, and abnormal hemodynamics; and carefully protecting the myocardium and brain. Since 2012, 41 patients (aortic dissection, 36 cases; arch aneurysm, 5) have undergone a procedure that was gradually modified, including the brain perfusion manner, branch priority, retrograde stent graft deployment via the femoral approach, a two-stage concept involving two-branch anastomosis. Arch replacement surgery could be completed within approximately 4 h, and during 2 years of treating aortic dissection or arch aneurysm, only four anastomoses were required during the first stage of surgery: two in the aorta and one each in the innominate and left common carotid arteries. No patient died of surgical causes, and no stent grafts deployed into the false lumen, which tends to occur with traditionally antegrade deployment.

**Fig. 1:**
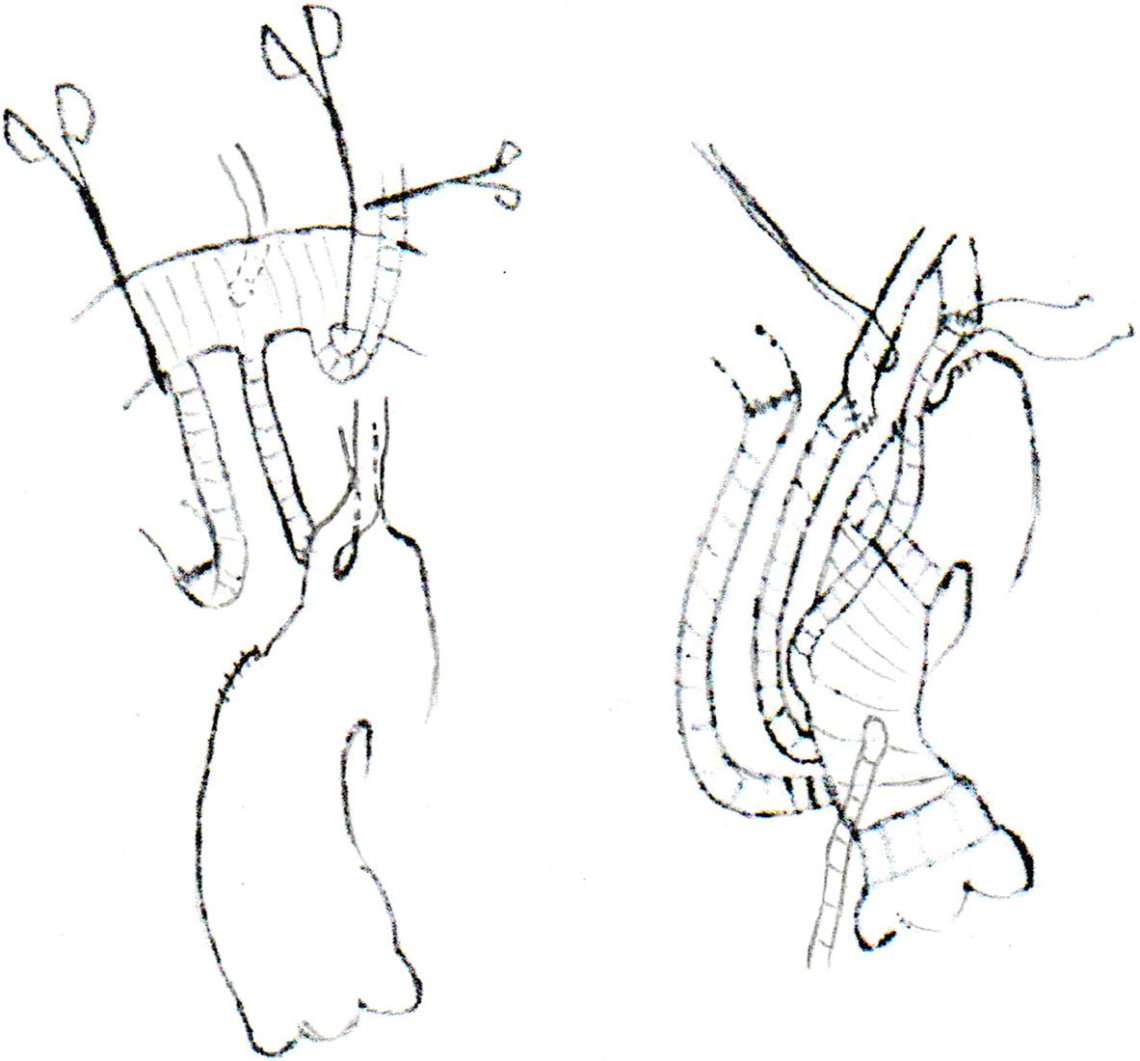
Showed how to dissect and expose the left subclavain artery in deep position. Left common carotid artery could be slung aside after transecting at the boot of the artery

The left subclavian artery remained perfused by the aorta from the true or false lumen after graft replacement after the first stage. It could be covered by the stent graft or reserved for the second stage. We were also able to reconstruct blood flow in all three supra-arch branches. For example, if a patient had an advantage of the left vertebral artery, we might consider an end-to-end anastomosis between the left subclavian artery and third graft branch during the rewarming period. The left carotid artery could be transected at the root and pushed aside to facilitate dissection of the subclavian artery (see Fig. 1).

Perfusion via the graft helps to avoid the deployment of other types of perfusion, e.g. auxiliary artery retrograde perfusion and antegrade cerebral perfusion via a catheter. Anastomosis of the supra-arch branches is performed before initiating CPB, and can thus be performed easily to ensure quality. The body temperature is lowered only to 28–32 °C during cardiac arrest, thus reducing the rewarming period. Altogether, these modifications save approximately 3 h.

If the left common carotid artery is occluded by a torn intima, the first anastomosis can be performed between the left subclavian artery and graft branch [3].

The very stiff guidewire and stent profile, which are curved prior to surgery, help to deliver the stent graft into the distal aortic graft.

**Fig. 2:**
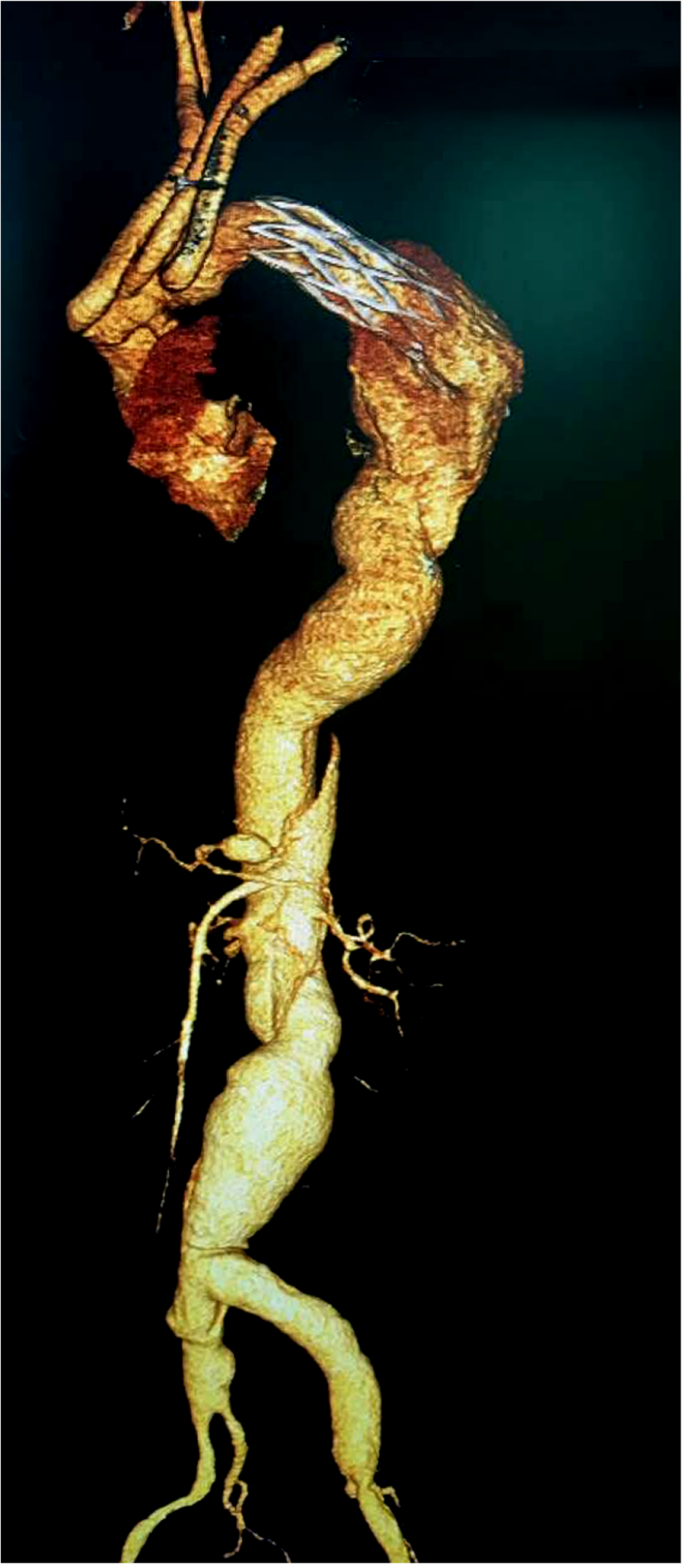
Showed a graft with a stented elephant trunk deployed antegradely into the false lumen of aortic dissection during circulation arrest. Stent graft deployed retrogradely is more efficient and safer for the descending aortic dissection than stented elephant trunk

**Fig. 3:**
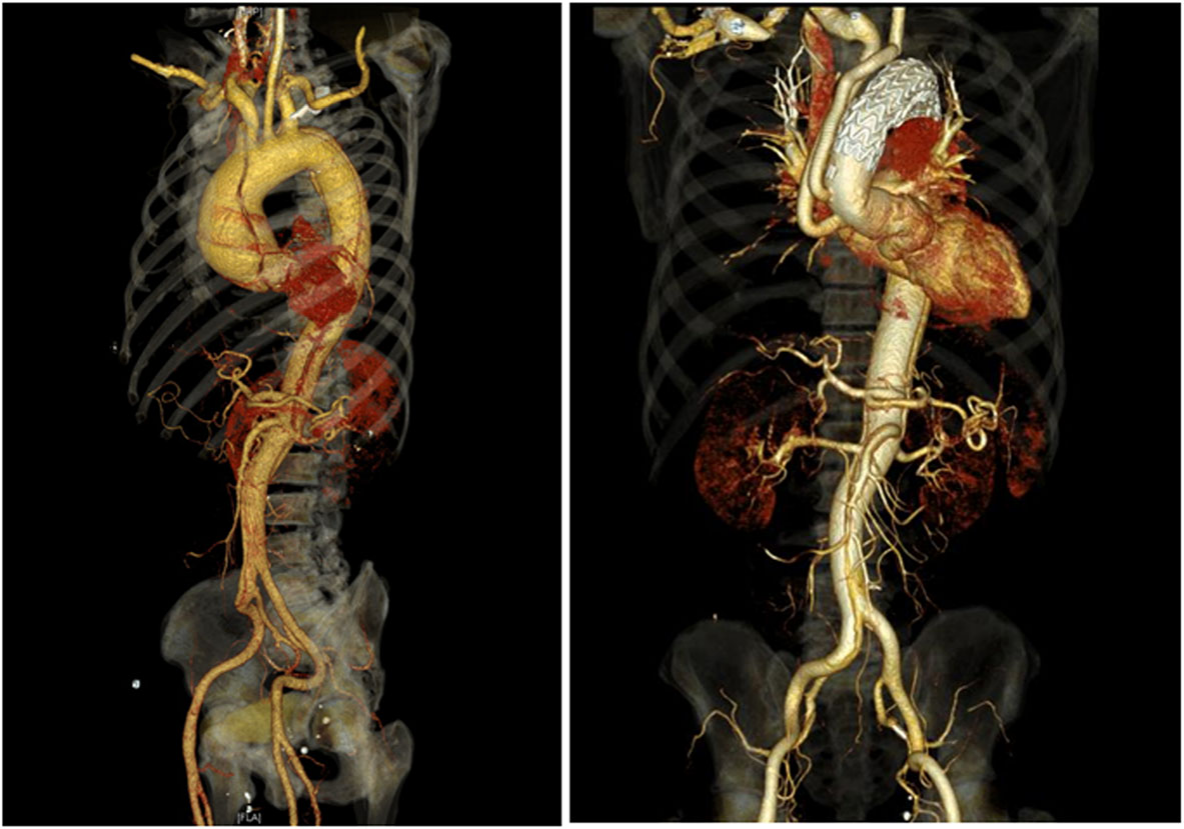
Showed the imaging of our modified procedure preoperatively and postoperatively, the stent graft was deployed retrogradely under the confirmation by angiography

For type A dissection, in order to shorten surgery time as possible, we recommend that our procedure be performed in two stages, especially for patients with the most severe conditions (e.g., hypotension with heart/brain/liver/kidney injury; older age; obese; cerebral infarction). However, one-stage treatment is required if intraoperative angiography (contract agent injected through the graft branch) confirms malperfusion of the viscera or lower extremities. The retrograde manner of stent deployment avoids plunging the stent graft into the false lumen through the torn intima (compare Figs. 2 and 3).

An arch aneurysm can be treated in a single stage, as false lumen deployment is not a concern. This is very similar to hemi-arch replacement with a stented graft/elephant trunk into the proximal descending aorta. In the former, we have more choosable specifications and models of the interventional stent grafts than the graft of the latter in China. That is important for protecting the descending aorta containing stent graft. The best stent graft we think is as soft as it can be to fit the diameter and shape of descending aorta. Further more, although aneurysm surgery is easier than dissection, we still suggest avoiding cardiac arrest if possible, unless the femoral approach of stent graft delivery is not permitted.

Although simplified, this procedure remains a major surgery for patients experiencing great distress, malperfusion, and abnormal hemodynamics resulting from an aortic dissection type A. If a sufficient segment of normal ascending aorta remains, a TEVAR procedure, which reserves the supra-arch branches, or a debranch procedure is recommended for simplicity and safety [4]. There remains room for potential improvements to further simplify this technique in the future.
